# Assessment of T cell immune responses towards HIV-1 epitopes designed by reverse immunogenetic approach: proof-of-concept in HIV+ Cameroonian children

**DOI:** 10.1186/1742-4690-9-S2-P239

**Published:** 2012-09-13

**Authors:** E Nemes, M Amicosante, F Ateba Ndongo, N Fainguem, S Moyo Tetang, A Bedin, E Temgoua, V Colizzi, G Cappelli

**Affiliations:** 1 Chantal Biya International Reference Centre for Research on HIV/AIDS, Yaounde, Cameroon; 2 University of Rome Tor Vergata, Rome, Italy; 3 Centre Mère et Enfant (Chantal Biya Foundation), Yaounde, Cameroon; 4 Higher Institute of Health (ISS), Rome, Italy

## Background

We tested whether 23 HIV-1 epitopes designed using a bioinformatics approach were recognized by HIV-infected children living in ethnically diverse Cameroon, which harbors a high HIV strain variety.

## Methods

Areas containing both promiscuous HLA-class I and II epitopes covering more than 90% of the HLA haplotypes present in the African population were identified within the most HIV-1 conserved sequence of each protein. Twenty-three peptides have been designed targeting gag (10), nef (6), tat (4), vpr (2) and vpu (1) proteins.

We enrolled 33 children vertically infected with HIV (age range: 3 months – 12 years), naïve for antiretroviral therapy, with CD4% >15% or CD4 count >350 cells/ml.

T cell recognition of single peptides was assessed by IFN-γ ELISpot. Pooled peptides (gag, nef, regulatory proteins) were used to assess helper (CD40L expression) and cytotoxic (CD107a expression on cell surface) functions, and proliferative capacity (CFSE dilution) by flow cytometry.

## Results

The majority (76%) of children recognized at least one peptide or peptide pool in at least one test, but none of the functional assays alone would have identified all responders.

Half (48%) of the peptides were recognized by at least one child in ELISpot assay, nef peptides being the most frequently targeted (by 67% of the responders). Three previously undescribed epitopes were identified, while 2 epitopes commonly considered immunodominant were not recognized.

In some children the same peptides were able to elicit different functions, while in other children diverse functions were induced by different peptides. The breadth of the epitope recognition and the number of different functions elicited were directly correlated and independent from the length of infection (age).

## Conclusion

These data provide a proof-of-concept for the rational design of T cell immunogens by reverse immunogenetic approach and support the parallel use of different functional assays for epitope mapping.

